# Comparing Skype (video calling) and in-person qualitative interview modes in a study of people with irritable bowel syndrome – an exploratory comparative analysis

**DOI:** 10.1186/s12874-019-0867-9

**Published:** 2019-11-29

**Authors:** Matthew Krouwel, Kate Jolly, Sheila Greenfield

**Affiliations:** Institute of Applied Health Research, University of Birmingham, 80, Hawkesley Mill Lane, Northfield, Edgbaston, Birmingham, B15 2TT UK

**Keywords:** Qualitative research, Qualitative methodology, Internet interviews, Skype, Data collection, Mode comparison

## Abstract

**Background:**

Within qualitative research in-person interviews have the reputation for being the highest standard of interviewer-participant encounter. However, there are other approaches to interviewing such as telephone and e-mail, which may be appropriate for a variety of reasons such as cost, time and privacy. Although there has been much discussion of the relative values of different interview methods, little research has been conducted to assess what differentiates them using quantifiable measures. None of this research has addressed the video call, which is the interview mode most like the in-person interview. This study uses quantifiable measures generated by the interview to explore the relative value of in-person and video call interview modes.

**Methods:**

Interview data gathered by a qualitative research study exploring the views of people with IBS about hypnotherapy for their condition were used. In-person and video call interviews using the same topic guide were compared on measures of length (time and word count), proportion of time the interviewer was dominant, the number of topics generated (codes) and the number of individual statements on which those topics were based.

**Results:**

Both interview methods produced a similar number of words and a similar number of topics (codes) were discussed, however the number of statements upon which the variety of topics was based was notably larger for the in-person interviews.

**Conclusion:**

These findings suggest that in in-person study interviews were marginally superior to video calls in that interviewees said more, although this was on a similar range of topics. However, the difference is sufficiently modest that time and budget constraints may justify the use of some video call interviews within a qualitative research study.

## Background

In-person face-to-face interviewing is often believed to be the ‘gold standard’ [1] in qualitive research, however, recent years have seen the rapid development of technologies which offer alternative interview modes, such as e-mail, instant messaging and video calling, as well as the increased use of older technologies such the telephone [2]. Each of these has its strengths and limitations, for example, e-mail allows for reflection before response, both by participant and interviewer, but equally this reduces spontaneity, making it appropriate when considered responses are sought but poor for getting the unfiltered truth [3]. By contrast, Instant Messenger (IM) which has a faster, more conversational pace than e-mail [4] is suggested to be good for interviews with groups who are uncomfortable with face-to-face communication, such as people with Autistic Spectrum Disorder (ASD), [5] however with IM there is a lack of body language and facial cues. As can be seen by these two examples, some approaches may be better than others in different circumstances and ultimately, the assumed superiority of the in-person interview is not demonstrably absolute.

One alternative to in-person interviewing is video calling. Video calls are an internet based technology which provides the synchronous experience of seeing and hearing the person at the end of the line, allowing for interviews to take place that are effectively face-to-face [6], arguably this is the closest current widely available technology comes to recreating the in-person experience whilst geographically separate. Skype, with over half a billion users at one time or another, [7] is one of the most well-known of these technologies. Video calling has many advantages as a research tool. It is cheaper and more time efficient than conducting in-person interviews. One study which used Skype to conduct job interviews in the place of in-person interviews estimated an average financial saving of $566.00 per interview and a total of 7 interviewer days saved over the project [8]. Video call interviewing also means that geographically hard to access participants, who might otherwise prove prohibitively expensive in time and effort to recruit and interview, can be reached [9, 10]. The cost and time savings are mirrored by a saving to the environment in emissions not generated by travel [11]. Further it has been noted that video calls are safer for both interviewer and participant as neither has to go to an otherwise unfamiliar location and some people prefer not to have their space imposed upon [11].

Video calls for qualitative interviewing have drawn consistent criticism for a number of reasons, including technical issues, such as time-lags on video and disconnected calls, [9–13] the need for participants to have the right software [14] and the latest version of that software [13]. The viewing perspective, referred to by Weller as the ‘talking heads’ perspective [15] may limit access to body language [16] although it is debatable how substantial this is [17]. It has been noted that video calls may reduce the interviews ability to reassure and comfort the interviewee when in distress through an inability to conduct such behaviours as passing tissues, or physical contact [18] although how appropriate the latter is may vary considerably with the circumstances. Additionally it has been argued that by having a poor view of the interviewee’s home the context of their life is lost, [15] although this may be equally true of in-person interviews conducted away from the home. The camera itself can be inhibiting to users, [9] as can the peculiar nature of making eye contact on most video call software which requires the users to look off centre to appear to be making eye contact, the result of the camera being at the edge of the screen [9]. Further, it is common to have a live image of oneself on the screen, encouraging you to monitor or talk directly to yourself [6]. Video call, by having two separate locations increases the chance of social interruptions from colleagues, family members or pets [9, 19]. It has been noted that certain populations may be excluded by the use of internet based technologies, [20] although this may decrease with time, [19] currently however 9% of the adult population of the UK state that they have never used the internet, most of them are over 55 years old and part of an observably declining trend [21].

The body of literature which compares video calling with in-person interviews is in its infancy. Several works have addressed the use of video calling for qualitative research [6, 9–11, 13, 15, 18, 19, 22]. Of these studies only two come from the healthcare sector, and these are both based on nurses experience, not patients [18, 19] meaning the possible effect of video call interviews upon patients’ responses is entirely unexplored. The literature includes assessments of postulated advantages and disadvantages [10, 18, 22] but predominantly consists of reflections upon the author’s experience of video calls as a research tool, [13, 15] five of the papers are based on studies in which both in-person and video call interview modes have been used [6, 9, 11, 18, 19]. These five papers focus upon topics such as rapport [9, 18, 19] and the logistical benefits and limits of video calls [6, 9, 11, 18]. As can be seen, theoretical differences have been well explored, but to date no attempt to test or quantify the impact of these differences has been made.

There is an established practice of using quantitative measures to assess differences between qualitative interview modes [23, 24]. Irvine [23] compared qualitative telephone and in-person interviews, using the duration of the interview and ‘dominance’, a measure of how long either the interviewer or interviewee were the dominant voice in the interview, as metrics. In-person interviews were found to produce longer interviews with the interviewee being the dominant speaker for more of the time [23]. Some quantitative work has been conducted on video calls as a qualitative interview tool, but thus far this has been limited to one study comparing video calls to telephone interviews amongst young adults, this found a lower take up rate but longer interview times amongst the video call population, [25] these results may be affected by the demographics of the study population and the state of the technology at the time. No research has yet been conducted directly comparing video calls with in-person interviews using quantitative measures. This paper compares the use of video calling and in-person interview modes in a qualitative research study using a variety of metrics, to assess if they produce similar or different volumes of data and topic variety, to provide guidance as to when video calling may be an appropriate approach to take.

## Methods

This study uses the transcripts from a study which used in-person and video calls with people who have refractory irritable bowel syndrome (IBS) [26], to identify their opinions of hypnotherapy as a treatment option for their condition. IBS is a functional disorder of the gut and digestion characterised by abdominal pain, constipation and diarrhoea [27]. It frequently leads to a number of behaviour changes, including socially inhibiting responses such as avoiding work situations, social situations and staying away over night for fear of a flare up of symptoms, [28] it is considered refractory if it has not responded to treatment after 12 month and an ongoing profile of symptoms has developed [29]. People with IBS may consider their illness to be an embarrassing topic [30] and as such a sense of safety and privacy with the interviewer and in the location of interview may be important. Hypnotherapy, the use of suggestion, imagery and metaphors in the hypnotic state to create change, has a demonstrable effectiveness in the treatment of refractory IBS [31] which is recognised by its inclusion within the UK’s National Institute of Health and Care Excellence (NICE) guidelines [29]. The source interview study received ethical approval under the University of Birmingham’s ethics procedures (reference ENR_15–1473).

### Methods of source study

A convenience sample [32] of UK resident adults who self-identified as having a formal diagnosis of IBS which had not responded to pharmacological treatments after 12 months and who had developed a continuing symptom profile [29] and who had never received hypnotherapy for their condition were recruited. Recruitment was via a poster campaign and by contacting IBS self-help groups and Facebook groups. No incentivisation was offered to potential interviewees but compensation for travel costs incurred in attending interviews was available. Both verbal and written consent for the interview were taken, in the case of video interviews verbal consent was obtained prior to the interview and confirmed in writing by post.

Interviews were conducted either in-person or face-to-face via video call. The decision to use mixed interview modes in this piece of research was taken whilst the study was ongoing and was in response to a sudden recruitment influx from internet advertising (Facebook). It was judged important to capitalise upon this influx rapidly due to the possibility of loss of interest by potential interviewees as the result of the time lag.

The transcription started from the point on the interview when the first question was asked by the interviewer. It concluded when the interviewer turned off the recording device, which was done when the answer to the last question was given and the interviewer judged that the interviewee had finished on the topic. Preamble and postamble were unrecorded. The interviewees perceptions of the interview process were not actively sought. Short pauses in speech were not recorded in the transcript, however if a pause was deemed to be unusually long or to denote a higher than average amount of thought an ellipse was inserted. Laughter and audible sighing were recorded with a single word within the transcript but no notes on body language were included.

A two-stage process of coding was undertaken. This process started with open coding [33]. Open coding involves a close read of the transcript to identify all statements, which are assigned a code. During open coding, codes are generated to fit the statements identified. For example, the statement “it’s got to the point that I know that whenever I’m eating out I know that I‘m going to swell” might generate the code ‘triggers for IBS’, and any subsequent statements regarding ‘triggers for IBS’ would then be assigned to this code. In this way 127 codes were generated. The second stage of the process was to reduce the codes by excluding any not relevant to the topics of interest, for example codes such as ‘non-IBS Life story’, and then amalgamating similar codes into a single code, so ‘massage’, ‘acupuncture’ and ‘meditation’ may all be combined under ‘complementary and alternative medicine (CAM)’. This left 79 codes. These transcripts were then coded again this time using the 79 codes only.

The same topic guide was used for both video call and in-person interviews. The same interviewer (MK) conducted and transcribed all the interviews. The idea to conduct the analysis of the two interview modes did not occur until after the coding had been undertaken. The full protocol of the study is available [26] as is the full source study [34].

### Methods of the study

#### Analysis

Six quantitative measures were used to assess the relative effect upon interviews of the mode of interview, these were: duration of interview in minutes, word count, speech rate, number of codes, number of statements and dominance. These were used to provide quantifiable data over a spread of measures. Two of these measures, duration [23, 25] and dominance, [23] have been used previously to assess the difference between interview modes. The addition of word count provides a balance to any potential biasing effect to the duration caused by the mode of interview, which if present would be highlighted by speech rate, a measure derived from word count and duration. There is no established practice of assessing the comparative depth and breadth of different qualitative methods, to be able to do this would help to identify some possible subtle impacts created by the different interview modes. To this end both the number of statements and the number of codes are used to act as proxy measures of depth and breadth respectively. An examination of the distribution of the word count data showed a skewed distribution and as such data is presented using the median. Excel 365 was used for calculations of totals and averages. Because of the small sample size no attempt to establish statistical significance was made.

#### Duration

Duration [23, 25] is a measure of the length of the transcribed portion of the interview in minutes, rounded to the nearest full minute. This provides a direct measure to compare the length of in-person and video calling interviews.

#### Word count

Word count is the total number of words said by both the interviewer and the participant. Word count provides a measure of how much is said in the interview, which may be different from the overall duration of the interview as some people will speak faster and slower or may take longer pauses [35].

#### Speech rate

Speech rate is a secondary measure calculated by dividing word count by duration to get the average number of words spoken per minute by both interviewer and participant. It is intended to identify whether the use of video calls effect the speed at which people express themselves.

#### Number of codes

A code is the designation applied to any number of comments in a transcript during the analysis phase which are under the same broad topic. There is a tradition of using the number of codes as a quantitative measure within content analysis [36] but this has not previously been used to compare modes of interview. The number of codes used on a transcript shows how much variety of discussion is present in that transcript, as such codes can be argued to be a measure of the material’s breadth of content, the more codes are present the greater breadth of material. Two levels of coding exist, the initial open coding and the second level of coding which is derived by reducing the initial open codes through removal of topics irrelevant to the aims of the study and by amalgamating similar codes, from here on this second stage of coding will be referred to as the amalgamated codes. The amalgamated codes are applied to the transcripts and will only record material which relates to those codes, meaning that everything recorded should be relevant to the area of interest to the study. This results in a set of codes which represent the range of discussion within the specific area of interest.

#### Number of statements

This is the number of statements relating to a code, it is a measure intended to give an idea of the depth and variety captured within the interviews. By using the number of statements as an indicator of how many different ideas or how much additional information was provided on a single code in this by the participants. In practice this means the code ‘ideal therapist’ may encompass multiple statements such as “they’d be able to provide evidence of qualifications” or “someone fairly sort of clean cut”.

As the transcriptions were analysed statements were highlighted and either assigned to an existing code or a code was generated for them. This was done within the Nvivo software package so the number of statements was recorded as analysis was conducted. This figure is distinct from the codes as a single code may have multiple statements in support of it e.g. 40 different codes to summarise the topics of 175 separate statements.

Broadly this metric can be said to represent the number of distinct comments made on a single topic. However, it is imperfect, there will be some instances where multiple comments on a point have been captured within a single statement as they are delivered within the same brief statement and conversely the same point having been made by the same participant multiple times at some remove from each other and thus have been recorded as multiple statements. The use of a single researcher for coding of the transcripts (MK) who was at the time of coding unaware of the statements ultimate use for this purpose will have meant a continuity of style across both in-person and video call interviews which is likely to standardise the error rate.

As statements are being used as a proxy for breadth and variety within the findings it is assumed that there will be only a correlation with the trend, rather than an absolute reflection of it, i.e. higher numbers of statements are likely to suggest more depth and variety but not give a precise indication of how substantial that is, as such small differences in cannot be taken to be meaningful, only large ones. This measure has yet to be validated over multiple studies or in the context of other potential metric or assessments of depth.

#### Dominance

Dominance is a measure of the percentage of the interview that the interviewer is leading [23], this is a subjective measure but quantifiable none the less. Kvale observed that qualitative interviews are not inherently equitable and that the very dynamic of a researcher posing questions for a participant to answer was indicative of a domination [37]. Transcripts of the interviews were analysed to identify verbal dominance within the interviews. Irvine’s definition of ‘floor holding’ was used to identify when the researcher was dominant, meaning that they were steering the exchange in some way or providing a summary, evaluation or assessment of the participant’s speech [23]. The transcript was examined and all sentences by the interviewer which contained an element judged to fit the ‘floor holding’ criteria were copied to a separate file. Any small utterings, for example an ‘ok’ or a ‘go on’ which may have prompted the participant to continue but did not alter the direction of talk have been discounted. The number of words used whilst dominant by the interviewer has then been calculated as a percentage of the total words within the interview, giving a percentage of interviewer’s dominance within the exchange [23].

## Results

### Participants

17 people completed an interview. One was removed from this analysis due to being asked an additional question regarding video call hypnotherapy, this being the question which prompted the idea for this analysis it was deemed inappropriate to include it as the interview and coding were conducted with an awareness of how the data may be used for this interview comparison. Additionally, there were questions of how the addition of a question may affect the character of an interview beyond the words directly attributable to that question. This left 16 interviews based on the original topic guide, 8 interviews were in-person, 8 via video call (Table 1). The average age of the two groups was comparable however, there were differences in the age range, ethnicity, gender composition and duration since first diagnosis (Table 1). Of the in-person interviews, one opted to do this at their home, five took place in private rooms at a University, two in other indoor public spaces. All the video call interviews appeared to take place in the participants’ homes, providing a modest window into their lived context. During the video call interviews two dropped calls occurred and one participant had to upgrade their software. In person interviews cost an average of £6.88 (range £2.50 – £32.30) in travel, video call interviews had no financial cost.

**Table 1:** characteristics of study participants

	In-person (range)	Video call
Sex (% Female)	75%	100%
Age in years (mean)	38.3 (22–63)	36.5 (26–43)
Ethnicity (% identify as white)	75%	100%

### Duration of interview in minutes, word count, speech rate

In-person interviews were 33% longer and used 14.6% more words, (Table 2). The speech rate was 16.2% higher for video calls (Table 2). At some point after transcription one of the recordings (0007) became corrupted and as such it could not be included in calculations of duration of interview, meaning only 15 interviews were used for this part of the analysis, however it was included in all the other analyses.

**Table 2:** The difference between in-person and video call interviews by number of words, duration and speech rate

	In person interviews	Video calling interviews	Difference	% difference between highest and lowest
Median number of words spoken	5451	4758	693	14.6
Range of words spoken	3825–8414	3879–6914	n/a	
Median interview time (minutes)	40	30	10	33.3
Range of interview time (minutes)	32–55	27–48	n/a	
Median speech rate (words per minute)	136	158	22	16.2
Range of speech rate (words per minute)	126–153	126–157	n/a	

### Number of codes, number of statements

The number of codes was similar for both the open coding group of codes and the amalgamated coding group of codes (Table 3), suggesting a similar breadth of topic was achieved by both approaches. However, the number of statements on which those codes were based for both open coding and amalgamated coding were higher for the in-person interviews (Table 3) suggesting that the in-person interviews generated a greater depth of discussion.

**Table 3:** The difference between in-person and video call interviews by number of codes generated

Open coding data	In person interviews	Video calling interviews	Difference	% difference between highest and lowest
Open coding data Nodes (median)	39	41	2	5.1
Open coding data statements (Median)	107	86.5	20.5	23.7
Secondary coding data nodes (Median)	36	35	1	2.9
Secondary coding data statements (Mean)	105	88	17	19.3

### Dominance

The interviewer was dominant for a greater proportion of the interview in the in-person interviews (30.0% by word count, see Table 4). When the interviewer’s dominant words were removed the difference between the words said by the interviewees was still higher for the in-person interviews (10.1% difference, see Table 4), however this is substantially lower than the difference in the overall word count (14.6% see Table 2).

**Table 4:** The difference between in-person and video call interviews by % of interview dominance

	In person interviews	Video calling interviews	Difference	% difference between highest and lowest
Median interviewer dominance (words)	1390	1069	321	30.0
Median interviewer dominance (%)	25.5	22.5	3	13.3
Median number of words (excluding interviewer dominance)	4061	3689	372	10.1

## Discussion

This comparison of in-person interviews with video call interviews identified that both produced a similar volume of data (words) and a similar breadth of topics (codes). However, in-person interviewees tended to make more individual points (statements) about those topics.

Upon examination of the data it becomes apparent that the full transcript word count and the duration of the interview were of minor importance. Equally interviewer dominance, as a percentage difference of the overall length of the interview, is only 3% and tells us little, however it does allow for us to adjust the overall word count to represent the words said by the interviewee alone. This adjusted figure was slightly higher for the in-person interviews over the video interviews (10.1%) which for a sample of this size is arguably negligible. However, the difference in word count observed after interviewer dominance was removed may be explicable by the relative lengths of time since first diagnosis, which is on average 6.4 years higher for the in-person group. The longer a person’s experience of living with IBS the more they are likely to have to say when recounting their experience of being diagnosed, attempting multiple treatments with varying degrees of success, different encounters with and reactions to various clinical situations and how over time it has impacted upon their life. These topics comprised a substantial proportion of the interviews. Either way it should be noted that the ‘Gold standard’ of interviewing (in-person) [1] did generate more words.

When examining the quality of those words, arguably the most important point, it was apparent that the number of codes used in the open coding and the amalgamated coding was almost identical. This strongly indicates that both methods produced a comparable breadth of understanding. However, the number of statements on which those codes are founded was quite different, being 23.7 and 19.3% higher for the in-person interviews, open coding and amalgamated coding respectively. This appears to suggest that for these interviewees at least there was a greater spread of distinct opinions, insight and viewpoints expressed within the topics by the in-person group, even if they did not move far from the core point of discussion. The greater number of statements will in some way be related to the higher number of words expressed by the in-person group, but as the number of statements was much higher (23.7 & 19.3%) than the additional number of words (10.1%) exactly what that relationship is remains unclear.

People involved in video call interviews used higher rates of speech (speech rate 16.2%). This was possibly due to some heightened anxiety or pressure brought about by the mode of interview. However, all participants in the video call interviews were calling from their home environments which could be considered innately more relaxing than being in a public or an unfamiliar location, particularly true when the sensitive nature of the topic is considered [30] and potentially the need to be close to lavatory facilities [38]. Another possibility is that it was an effect of the ‘forward leaning’ position which sitting at a computer at a desk or table promotes, this position is known to induce changes in breath, [39] and thus impact upon speech [40].

### Reflections

From a qualitative perspective the researchers neither experienced nor noted any consistent difference between the nature and character of the interviews by mode. Even rapport which some have anticipate as being inhibited by the camera [9] did not appear to be different, it should be noted that the interviewer (MK) is experienced at using skype to conduct patient work and as such entered the interviews comfortable with the mode. There were however a few points not covered by the quantitive analysis which are worthy of discussion.

As noted, video calls have drawn considerable criticism for technical issues [9–13] and a few technical issues occurred during the study, such as dropped calls and frozen screens. However, the interviewer found that rather than being barriers to rapport, sorting these issues out became a bonding exercise possibly due to the vulnerability [41] which both parties experienced as they shared their mutual lack of technical expertise.

It has been suggested that because video calls provide only a very limited window into an interviewee’s home when compared to a home visit that there may be a loss of contextual understanding of that person’s life by the interviewer [15]. However, as all but one of the in-person interviews took place away from the home and all the video call interviews appeared to be from the interviewee’s home, ultimately video calls may prove superior to in-person interviews with regard to getting some insight from the interviewee’s lived context.

As observed by other users of video call interviews they allow for substantial savings in time and cost [8, 9] and this was the experience on this study. In this study video calls were made using a software package which was already available to the researchers, incurring no additional cost. The cost of the in-person interviews in travel was minimal, but this reflects the limited geographical area of the in-person interviews (Midlands region, UK). However, several of the video interviews involved interviewees who lived hundreds of miles away from the interviewer and would have necessitated air or sea travel to reach in-person. The process of capturing these interviews would have substantial cost implications in travel and accommodation. The primary researcher, a PhD student, was giving their time for free, as such travel time did not impact upon costs and because the researcher travelled to participants chosen location none of the participants requested travel costs. Savings in cost and time would allow for qualitative research to be conducted within quantitative trials without undue pressure upon the overall budget providing greater understanding and context of the quantitative findings [42].

### Strengths and limitations

The study was conceived after the initial collection and transcription of the data for the original IBS study from which the data was taken, as such researcher bias, something considered a major potential issue in qualitative interviews, [43] is unlikely to have affected the initial data. However, it is an exploratory study only with a modest population and no randomisation and as such further research is required.

There was heterogeneity between the two groups, with the in-person group containing both male participants and all the Black, Asian and Minority ethnic (BAME) participants. The age distribution of the participants is noteworthy with all the older (≥45 years) and younger (≤25 years) interviewees participating in in-person interviews whilst most video call interviews came from those in their 30s and 40s. This could in part be the result of the use of Facebook as part of the recruitment strategy, in the UK Facebook has an average user age of 40, [44] and all the people in their 30s and 40s, including the one in-person interviewee, were recruited via it, whereas the younger and older participants all came through posters and word of mouth. Facebook’s average user age is notably older than some other social media [44] which suggests that a variety of social media platforms should be used to recruit a more varied population for any study looking to use the internet as part of its recruitment strategy. Our findings appear to support the notion that older people may be inadvertently under represented when internet recruitment and interview strategies are employed [20] and as such more traditional recruitment methods, such as posters, and in-person interviews should be present when these groups are desired to be a part of a study population.

## Conclusion

This study found that in-person interviews were slightly superior to video calls in that they produced more words and substantially more statements in support of a similar number of codes. However, the difference was modest, and video call interviews could offer substantial savings of time and budget. As such the use of video call interviews may be justifiable in situations where otherwise the research would not be possible, for example with rare diseases where the population may be highly dispersed or there are situations which are dangerous to enter. In-person interviews should be preferred where older populations are sought due to relatively low levels of familiarity with the technology. Ultimately a mixed mode of interviewing with some interviews being conducted in-person and the costliest in time or money or potentially danger being conducted by video call may be the most efficient balance.

## Data Availability

The datasets used and/or analysed during the current study are available from the corresponding author on reasonable request.

## Abbreviations

ASD: Autistic Spectrum Disorder
CAM: Complementary and Alternative Medicine
IBS: Irritable Bowel Syndrome
IM: Instant messenger
NHS: National Health Service
NICE: National Institute of Health and Care Excellence
NIHR: National Institute for Health Research
UK: United Kingdom
